# The validation of a home food inventory

**DOI:** 10.1186/1479-5868-5-55

**Published:** 2008-11-04

**Authors:** Jayne A Fulkerson, Melissa C Nelson, Leslie Lytle, Stacey Moe, Carrie Heitzler, Keryn E Pasch

**Affiliations:** 1 School of Nursing, University of Minnesota, Minnesota, USA; 2 Division of Epidemiology & Community Health, University of Minnesota, Minnesota, USA

## Abstract

**Background:**

Home food inventories provide an efficient method for assessing home food availability; however, few are validated. The present study's aim was to develop and validate a home food inventory that is easily completed by research participants in their homes and includes a comprehensive range of both healthful and less healthful foods that are associated with obesity.

**Methods:**

A home food inventory (HFI) was developed and tested with two samples. Sample 1 included 51 adult participants and six trained research staff who independently completed the HFI in participants' homes. Sample 2 included 342 families in which parents completed the HFI and the Diet History Questionnaire (DHQ) and students completed three 24-hour dietary recall interviews. HFI items assessed 13 major food categories as well as two categories assessing ready-access to foods in the kitchen and the refrigerator. An obesogenic household food availability score was also created. To assess criterion validity, participants' and research staffs' assessment of home food availability were compared (staff = gold standard). Criterion validity was evaluated with kappa, sensitivity, and specificity. Construct validity was assessed with correlations of five HFI major food category scores with servings of the same foods and associated nutrients from the DHQ and dietary recalls.

**Results:**

Kappa statistics for all 13 major food categories and the two ready-access categories ranged from 0.61 to 0.83, indicating substantial agreement. Sensitivity ranged from 0.69 to 0.89, and specificity ranged from 0.86 to 0.95. Spearman correlations between staff and participant major food category scores ranged from 0.71 to 0.97. Correlations between the HFI scores and food group servings and nutrients on the DHQ (parents) were all significant (p < .05) while about half of associations between the HFI and dietary recall interviews (adolescents) were significant (p < .05). The obesogenic home food availability score was significantly associated (p < .05) with energy intake of both parents and adolescents.

**Conclusion:**

This new home food inventory is valid, participant-friendly, and may be useful for community-based behavioral nutrition and obesity prevention research. The inventory builds on previous measures by including a wide range of healthful and less healthful foods rather than foods targeted for a specific intervention.

## Background

Availability of foods in the home has been shown to be significantly associated with dietary practices, intake, and eating patterns [1-5]. Although this area of research has been growing and several instruments have been developed to assess the home food environment (see [6] for a comprehensive review), often the instruments include a limited number of items selected to address a specific aim such as fruit and vegetable availability for cancer prevention or high-fat food availability for cardiovascular health. Instruments that provide a more comprehensive assessment of food and energy availability in the home are currently unavailable. Given current scientific interest in the contextual and environmental influences on energy balance and obesity, there is a great need for such inventories.

When developing such assessment inventories, thorough evaluation is needed, including several dimensions of validity testing. *Criterion validity *tests the performance of an instrument by comparing it to a gold standard [7]. A rigorous but logistically-challenging method for assessing criterion validity for a home food inventory is to require participants and study staff to independently complete a home food availability instrument, and then compare their responses using the staff report as the "gold standard." Comparisons are evaluated for consistency of results between the two methods using kappa or correlation statistics. In addition, comparisons may be made by examining sensitivity (i.e., proportion of foods in the home assessed as present by the staff that were accurately identified as present in the home by the participant) and specificity (i.e., proportion of foods in the home assessed as absent by the staff that were accurately identified as absent in the home by the participant). *Construct validity *is suggested when expected relationships are shown between the measure and other variables in a conceptual framework [7]. For example, if an HFI shows high availability of high fat foods in the home, one might expect family members to report a high calorie intake in their diet.

The development of many of the home food availability measures described in the literature include a very limited number of foods, and have not included comprehensive validity testing, particularly criterion and construct validity. Criterion validity is very important if participants in research studies are expected to complete self-report home food inventories on their own while in their homes. Construct validity is also important because an instrument should be associated with expected health outcomes. Only four studies have demonstrated criterion validity of home food inventories [2,8-10], and only two have demonstrated construct validity [2,8], and each has its limitations (as described below).

Crockett and colleagues [8] developed a shelf inventory of 80 foods in 12 categories based on foods targeted in a cancer risk reduction program. Only perishable foods targeted in the intervention program were included on the inventory. They conducted two criterion-related validation studies that compared participant and staff-reported inventories. In the two studies, sensitivity was reported as 0.86 and 0.87, and specificity was reported as 0.92 and 0.90, respectively. In addition, Cohen's kappa showed significant overall agreement (p < .0001) between participant and staff reports in both studies. Furthermore, construct validation was assessed by comparing inventory responses with food frequency questionnaires, with overall agreement of 73.6%

Miller and Edwards [9] assessed the face, content and criterion validity for a 166-item shelf inventory that was based on previous work [8,11] with modifications for fat- and sugar-modified foods that would be relevant to the purchases of individuals with diabetes. Similar to the previous inventory, mostly perishable foods were included. Thirty-one older adults diagnosed with Type 2 diabetes completed the inventory and within 48 hours study staff directly observed foods in the home. Cohen's kappa statistic was 0.87 and sensitivity and specificity were 0.90 and 0.97, respectively. Construct validity was not assessed.

Marsh and colleagues [10] conducted a study with 48 parents that demonstrated criterion validity for a 34-item fruit, juice, and vegetable availability questionnaire. Parents reported the presence of these foods in the home within the last seven days, and staff conducted inventories on the same visit. In addition to perishable foods, frozen, canned and dried fruit/vegetable products were included. Cohen's kappa values ranged from 0.24–0.53 for juices, 0.12–0.76 for fruits, and 0.22–0.66 for vegetables. Overall sensitivity and specificity values for total fruit, juice and vegetables were 36.8 and 39.1, respectively. Construct validity was not assessed.

Lastly, Raynor and colleagues [2] completed a type of criterion validity and construct validity of a household food availability instrument (22 high-fat and 22 low-fat items) with 165 adults. Criterion validity was accomplished by correlating the number of high-fat and low-fat food items reported by two adults in the same household (r = 0.69, p < .001 and r = 0.59, p < .001, respectively). However, gold standard criterion validity comparing participant report to trained staff report was not completed. Construct validity considered correlations between availability of high-fat foods and low-fat foods with fat consumption via a food frequency questionnaire, and showed significant (p < .001) correlations (r = 0.25 and r = -0.33, respectively).

In comparison to other published home food inventories [6], these four studies represent the most comprehensive methods that have been used to test validity to date. Despite their relatively rigorous validity testing, however, only two studies demonstrated construct validity, and each of these measures is somewhat limited in scope, with none providing a comprehensive assessment of overall home food and energy availability. Thus, given the lack of comprehensive home food inventories that have been rigorously validated, there is a need for additional instrument development in this area. An inventory assessing a wide range of food exposures in the home, including both healthful and less healthful foods, may be important in understanding contextual influences on obesity, weight gain, and nutritional intake. In addition, such an inventory could be useful in determining appropriate intervention strategies that may fit the needs of individual households or might identify targets for public health messages. The purpose of the present study was to develop and validate a home food inventory that is easily completed by adults in their homes and includes both healthful and less healthful foods as well as reduced-fat and regular-fat varieties of foods potentially related to the obesity epidemic.

## Methods

### Procedures, Participants, and Measures

The University of Minnesota's Institutional Review Board approved this study, and all participants signed the appropriate parental consent and student assent forms. The study was conducted in three phases. First, brief field testing of the newly developed HFI was conducted with a small sample of adults (n = 5) to assess ease of completion and comprehension as the participants indicated which foods were difficult to inventory. The inventory food list was re-evaluated at this stage and foods were added if participants frequently indicated that there were foods to code without a place to do so on the inventory. Second, adults in the community were recruited to complete the home HFI in their homes while allowing trained staff members in their homes to complete the inventory independently (criterion validity testing with Sample 1). Third, parents and students were recruited for participation in the IDEA (Identifying Determinants of Eating and Activity) study [12] in which parents completed the HFI and Diet History Questionnaire (DHQ) and students participated in 24-hour recall dietary interviews (construct validity with Sample 2).

#### Sample 1

For the criterion validation phase, 51 adults were recruited from 19 area Minneapolis Park and Recreational Centers via posted flyers. Trained research staff traveled to the participants' homes to complete consent procedures and complete to the HFI. Although participants and staff completed inventories at the same time, they began their assessments in different parts of the home and were instructed not to communicate with each other as they completed the inventory. Participants were provided with a $30 gift card for their participation.

#### Sample 2

For the construct validation phase, 349 families (one student between the ages of 10 and 17 years and one parent/guardian or other adult caregiver) were recruited from the following sources: 1) an existing cohort of youth participating in the Minnesota Adolescent Community Cohort (MACC) Tobacco Study [13], 2) a Minnesota Department of Motor Vehicle (DMV) list restricted to the seven-county metro area, or 3) a convenience sample drawn from local communities. Of the 349 youth/adult pairs measured, 26% were recruited from the MACC cohort, 49% were recruited from the DMV sample and 25% were recruited from the convenience sample [12].

In the larger IDEA study, youth and adults pairs scheduled a visit to an IDEA clinic where anthropometric measures were taken and psychosocial surveys (that included demographic characteristics) were administered to both students and adults. Instructions for additional measures were given at this time and parents received a packet of instruments to take home to complete and return by mail. Included in this packet were the HFI and the DHQ. The DHQ is a food frequency that has been widely used with adults (NCI). Students were told to expect that three dietary recall interviews would be conducted with them by telephone within the next month, and were provided with a two-dimensional food model packet to help them estimate portion size. The final sample for construct validation includes data from the 342 families who completed the HFI, DHQ and dietary recalls (98% of sample).

### Measures

#### Home Food Inventory

To develop the initial set of food items, the investigators examined existing instruments [8,14] and reviewed the literature that identified major contributors to overall energy intake (e.g., [15]). This process allowed us to evaluate the foods listed in inventories that were developed for a specific limited purpose (e.g., a diabetic population, nonperishable foods, etc) as well as expand the items in our inventory to include foods known to be associated with energy intake in the population. In addition, based on literature that demonstrates a high correlation between readily accessible foods (i.e., foods in plain view) and their intake [3], two items were added to assess the accessibility of healthful foods within the main kitchen area and the refrigerator. Thus, we evaluated the literature and instruments to date, and added foods that provided a more comprehensive inventory of foods associated with dietary intake of adults in the US.

During the course of initial validity testing (Sample 1), changes in the number of items and instructions occurred. For example, participants were allowed to "write in" foods within given categories (e.g., dairy, fruits, vegetables), and if "write in" responses were found to be frequent, they were added as items on the inventory. During the validity testing with Sample 1, the HFI had 186 items while the final inventory administered to Sample 2 included 190 items.

HFI items are listed in a checklist type format with yes/no (1/0) response options. Higher scores represent greater availability. In addition, participants were instructed to check whether the vegetable, fruit, and bread items were fresh, frozen, dried or canned, as appropriate. The category order was set up to facilitate ease of completion, beginning with the refrigerated items, followed by frozen items, and non-perishable items. Participants are instructed to look for these foods in all areas of the home where food is stored, including the refrigerator, freezer, pantry, cupboard, and other areas (e.g., basement). Participants were informed that lower fat products may be labeled as "reduced-fat," "low-fat," "light," "nonfat," or "skim." Foods in the dairy, added fats, frozen desserts, prepared desserts, and savory snacks were categorized into regular-fat or reduced-fat groupings; beverages were categorized into regular sugar and low sugar categories; and foods in the two ready-access categories were further subgrouped into healthful and less healthful categories. Although the categorization of foods into healthful and less healthful categories may not be entirely straightforward, we assessed each food by its typical fat and sugar content when determining its category. To assess the overall obesogenic home food availability, a summative score was created that includes regular-fat versions of cheese, milk, yogurt, other dairy, frozen desserts, prepared desserts, savory snacks, added fats; regular-sugar beverages; processed meat; high-fat quick, microwavable foods; candy; access to unhealthy foods in refrigerator and kitchen. The obesogenic home food availability score potential range was from 0–71 (present sample: range = 9–53, M = 29.4, SD = 7.6). The HFI can be requested from the primary author. A table reflecting which foods are included in each food group/subgroup is provided in Additional file 1. The inventory took approximately 30–45 minutes to complete depending upon the amount of food stored in the home.

#### 24-Hour Recall Interviews

Students in Sample 2 completed three telephone-administered 24-hour dietary recalls following their clinic visit (response rate for students completing three recalls was 86%). Dietary recalls were conducted for two weekdays and one weekend day, with the aim of having each of the three recalls completed within a 2-week period. In general, multiple dietary recalls are widely accepted as a valid and reliable method for dietary assessment, and have yielded acceptable validity in children as young as 10 years [16]. Trained and certified staff from the Nutrition Coordination Center (NCC) at the University of Minnesota administered the recalls, using the Nutrition Data System Research (NDS-R) software [2006, Nutrition Coordinating Center, University of Minnesota, Minneapolis, MN] with an interactive, interview format with direct data entry linked to a nutrient database [17]. The NDS-R data set allows for the examination of both nutrient intakes and food group information (e.g., servings of fruits and vegetables consumed).

#### Diet History Questionnaire

Dietary assessment for parents was conducted using the Diet History Questionnaire (DHQ) food frequency instrument developed by the National Cancer Institute (NCI). Parents received the DHQ at the clinic visit and were asked to mail it in when completed. This instrument consists of 144 food items and includes both portion size and dietary supplement questions. Requiring approximately one hour to complete, it has been widely used to characterize usual food and nutrient intakes in numerous adult populations.

Several studies have been conducted to assess the validity and calibration of the DHQ. Findings indicate that the DHQ provides reasonably valid estimates for usual intake of most nutrients and that it performs as well or better than other well-known food frequency instruments available in the field [18-20]. The food list and nutrient database used for standardized analysis of the DHQ are derived using national dietary data from the US Department of Agriculture's Continuing Survey of Food Intakes by Individuals (1994–96) [20].

### Data Analysis

To assess criterion validity using data from Sample 1, participants' and research staffs' assessment of home food availability were compared. Consistent in research of criterion validity, the staff report was considered the gold standard as they were trained on how to use the inventory. Validity was evaluated by calculating kappa, sensitivity, and specificity between participant and staff reports on the presence of individual foods. To summarize these results, we calculated the average of these individual kappas across both major and minor food groupings. In addition, to test the performance of the instrument's assessment of broad food categories (rather than individual foods), we assessed the extent of agreement between food group summary scores between participant and staff reports by creating additive summary scores for major and minor food groupings (e.g., overall dairy score, cheese score, respectively). Using data from Sample 2, construct validity was assessed by examining Spearman correlations of five major food category scores on the HFI (i.e., dairy, vegetables with and without potatoes, fruit, and meats & other nondairy protein) with number of servings of the same foods as well as nutrients that should be correlated with these foods (e.g., calcium with dairy, Vitamin C with fruit) with foods and nutrients from the DHQ and 24-hour recall interviews. These five categories were chosen since it was possible to create similar categories across the three measures. In addition, we assessed construct validity of the obesogenic home food availability score by comparing it to both parental and adolescent reports of energy intake. All analyses were conducted in SAS (v9.1, SAS Institute, Inc., Cary, NC, 2003).

## Results

### Sample Characteristics

As shown in Table 1, Sample 1 consisted of 51 adults (predominantly female). About two-thirds of the sample was white, followed by African American, American Indian, Mixed race/ethnicity, Latino, and Asian. More than half of the sample had a college degree. Student gender for Sample 2 was equally distributed and most identified themselves as white. The majority of students reported living with their mother and father together. Students were primarily attending public schools, and more than two-thirds of the sample was in 9^th^–12^th ^grade. Parent participants in Sample 2 were predominantly white females with some college education.

**Table 1:** Characteristics of criterion validity sample (Sample 1) and construct validity sample (Sample 2)

	Sample 1	Sample 2	
**Characteristic**	Adults (n = 51)	Parents (n = 342)	Students (n = 342)

Gender, %			
Females	94.1	75.6	51.0
Education, %			
Less than HS	4.0	0.6	100.0
HS or GED	8.0	8.6	--
Some college or vocational school	26.0	26.1	--
College degree	28.0	35.2	--
Training beyond college degree	34.0	27.8	--
Race/ethnicity, %			
White	68.6	98.8	93.4
African American	13.7	0.3	1.4
American Indian	5.9	0.0	0.0
Asian	2.0	0.0	0.3
Hispanic/Latino	3.9	0.0	0.0
Mixed	5.9	0.9	4.6
Age (years; M, SD)	39.4	46.7 (0.3)	15.4 (0.1)

### Criterion Validity

Table 2 provides information regarding criterion validity with comparisons of reports of the presence of individual food items between the trained staff data (gold standard) and participants' data, including the number of items, the average kappa statistic, the average sensitivity value, and the average specificity value for each food category. In addition, Spearman correlations of major food categories comparing data from staff and participants are provided. The number of items in each major food category ranged from five to 26.

**Table 2:** Inventory major and subgroup food category validity indices (Sample 1, n = 51)

Food category	# Items	Average kappa for category	Sensitivity	Specificity	Staff/participant correlation^a^
**Dairy**	**21**	**0.72**	**0.81**	**0.91**	**0.92**
Cheese	11	0.64	0.74	0.90	0.85
Regular fat	5	0.62	0.76	0.84	0.70
Reduced fat	6	0.65	0.72	0.95	0.80
Milk/other dairy beverages	6	0.89	0.90	0.97	0.90
Regular fat	1	0.94	1.00	0.98	0.88
Reduced fat	5	0.87	0.88	0.97	0.89
Yogurt	2	0.71	0.85	0.86	0.89
Regular fat	1	0.70	0.78	0.91	0.70
Reduced fat	1	0.72	0.91	0.81	0.73
Other Dairy	3	0.78	0.87	0.90	0.87
Regular fat	2	0.73	0.90	0.86	0.78
Reduced fat	1	0.88	0.82	1.00	0.88
**All vegetables, including potatoes**	**20**	**0.80**	**0.89**	**0.90**	**0.88**
All vegetables, no potatoes	19	0.80	0.89	0.90	0.88
**Fruits**	**26**	**0.83**	**0.87**	**0.95**	**0.95**
**Meats & other nondairy protein**	**16**	**0.74**	**0.88**	**0.86**	**0.85**
Processed meat	4	0.74	0.84	0.91	0.78
All other protein	11	0.74	0.89	0.85	0.83
**Added Fat**	**13**	**0.76**	**0.84**	**0.92**	**0.79**
Regular fat	8	0.78	0.88	0.91	0.76
Reduced fat	5	0.72	0.78	0.94	0.77
**Frozen Desserts**	**7**	**0.64**	**0.70**	**0.94**	**0.71**
Regular fat	3	0.83	0.86	0.95	0.82
Reduced fat	4	0.50	0.58	0.93	0.70
**Prepared Desserts**	**8**	**0.61**	**0.69**	**0.93**	**0.73**
Regular fat	6	0.58	0.68	0.92	0.65
Reduced fat	2	0.81	0.80	0.98	0.81
**Savory Snacks**	**18**	**0.73**	**0.84**	**0.91**	**0.95**
Regular fat	10	0.71	0.88	0.89	0.93
Reduced fat	8	0.76	0.78	0.95	0.91
**Microwavable/quick-cook foods**	**8**	**0.71**	**0.78**	**0.95**	**0.81**
**Bread^b^**	**12**	**0.71**	**0.80**	**0.91**	**0.86**
Wheat	5	0.77	0.76	0.95	0.74
White	7	0.66	0.83	0.87	0.79
**Dry breakfast cereal^c^**					
Whole grain	1	--	--	--	0.75
High sugar	1	--	--	--	0.87
Low sugar	1	--	--	--	0.77
**Candy**	**5**	**0.79**	**0.87**	**0.94**	**0.97**
**Beverages**	**9**	**0.76**	**0.86**	**0.88**	**0.84**
Regular sugar	6	0.74	0.86	0.86	0.78
Low sugar	3	0.82	0.85	0.93	0.89
**Kitchen accessibility**	**12**	**0.74**	**0.74**	**0.95**	**0.75**
Access to healthy foods	6	0.66	0.63	0.95	0.67
Access to unhealthy foods	6	0.83	0.84	0.96	0.86
**Refrigerator accessibility**	**15**	**0.63**	**0.75**	**0.89**	**0.73**
Access to healthy foods	9	0.59	0.70	0.91	0.72
Access to unhealthy foods	6	0.68	0.81	0.86	0.73
**Obesogenic food availability score**	**71**	**0.73**	**0.83**	**0.91**	**0.94**

^a^Spearman correlation between staff and participant report of foods present in home.

^b^Pilot form did not include croissant item.

^c^Kappa statistic not available owing to multiple category response options for item.

For all 13 major food categories and the two accessibility categories, Cohen's kappa ranged from 0.61 (prepared desserts) to 0.83 (Fruits), sensitivity values ranged from 0.69 (prepared desserts) to 0.89 (vegetables), and specificity ranged from 0.86 (meat & other nondairy protein, bread, and beverages) to 0.95 (fruits, microwavable/quick-cook foods, and kitchen accessibility). All kappas assessing agreement between staff and participant reports of major food categories were greater than 0.60, indicating substantial agreement. Spearman correlations between staff and participant major food category scores ranged from .71 (frozen desserts) to .97 (candy). All correlations between staff and participant reports of major food group scores were greater than 0.70. All validity statistics for the obesogenic home availability score were equally acceptable.

### Construct Validity

Results for construct validity (correlations between five HFI major food categories and the same food categories and expected associated nutrients from the DHQ and 24-hour recalls) are presented on Table 3. All of the HFI major food category scores among the parents were significantly and positively correlated with the corresponding food group servings on the DHQ. Furthermore, Vitamin A, Vitamin C, fiber, and calcium were significantly and positively associated with their respective HFI major food categories (e.g., calcium with the dairy score, Vitamin C with the fruit score). Similarly, the obesogenic home availability was significantly and positively associated with parental energy intake. As shown in Table 4, significant, but attenuated, correlations were found between several of the HFI major food category scores and 24-hour recall nutrient intake values among the adolescents (HFI dairy score with recall dairy servings and recall calcium, HFI vegetable scores with recall vegetable servings and recall Vitamin A, and HFI fruit score and recall Vitamins A, C, and fiber). However, for nearly half of the food group categories and nutrients, dietary intake based on the 24-hour recalls was not found to be associated with availability of related products in the home. The obesogenic home food availability score was significantly and positively associated with adolescent energy intake.

**Table 3:** Correlations between HFI food categories and DHQ food serving and nutrient data (Sample 2, n = 342)

HFI major food category	DHQ servings and nutrients	Spearman Correlation	p-value
Total number of dairy products	Total number of dairy servings	0.15	<.01
	Average calcium (mg)	0.16	<.01
Total number of vegetables (including potatoes)	Total number of vegetable servings (including potatoes)	0.34	<.0001
	Vitamin A (IU)	0.27	<.0001
	Vitamin C (mg)	0.27	<.0001
	Fiber (g)	0.20	<.001
Total number of vegetables (excluding potatoes)	Total number of vegetable servings (excluding potatoes)	0.34	<.0001
	Vitamin A (IU)	0.26	<.0001
	Vitamin C (mg)	0.26	<.0001
	Fiber (g)	0.20	<.001
Total number of fruits	Total number of fruit servings	0.37	<.0001
	Vitamin A (IU)	0.26	<.0001
	Vitamin C (mg)	0.30	<.0001
	Fiber (g)	0.26	<.001
Total number of meats & other nondairy protein	Total number of non-dairy protein servings	0.23	<.0001
	Protein (g)	0.13	<.01
Obesogenic food availability score	Energy (kcals)	0.16	<.01

* Summative score that includes regular-fat versions of cheese, milk, yogurt, other dairy, frozen desserts, prepared desserts, savory snacks, added fats; regular-sugar beverages; processed meat; high-fat quick, microwavable foods; candy; access to unhealthy foods in refrigerator and kitchen.

**Table 4:** Correlations between HFI food categories and dietary recall food serving and nutrient data (Sample 2, n = 342)

HFI major food category	Dietary recall servings and nutrients	Spearman Correlation	p-value
Total number of dairy products	Total number of dairy servings	0.15	<.01
	Average calcium (mg)	0.13	<.05
Total number of vegetables (including potatoes)	Total number of vegetable servings (including potatoes)	0.16	<.01
	Vitamin A (IU)	0.13	<.05
	Vitamin C (mg)	0.10	.08
	Fiber (g)	0.06	.28
Total number of vegetables (excluding potatoes)	Total number of vegetable servings (excluding potatoes)	0.17	<.01
	Vitamin A (IU)	0.13	<.05
	Vitamin C (mg)	0.10	.08
	Fiber (g)	0.05	.34
Total number of fruits	Total number of fruit servings	0.07	.17
	Vitamin A (IU)	0.11	<.05
	Vitamin C (mg)	0.13	<.05
	Fiber (g)	0.17	<.01
Total number of meats & other nondairy protein	Total number of non-dairy protein servings	0.03	.56
	Protein (g)	0.02	.68
Obesogenic food availability score*	Energy (kcals)	0.13	<.05

* Summative score that includes regular-fat versions of cheese, milk, yogurt, other dairy, frozen desserts, prepared desserts, savory snacks, added fats; regular-sugar beverages; processed meat; high-fat quick, microwavable foods; candy; access to unhealthy foods in refrigerator and kitchen.

## Discussion

The present study describes the validation of a home food inventory designed to include a wide range of foods that contribute to energy intake, including more and less healthful foods. The wide range of foods included on the inventory speak to its content validity and study findings indicate substantial criterion, and construct validity for the new inventory, particularly for adults. In addition, the checklist type format is easily completed by research participants in their homes without undue response burden.

The demonstrated criterion validity of the new home food inventory as shown by high kappa, sensitivity, specificity values and high correlations between participants' and staffs' reports of foods in the home suggests that the instrument could be used effectively for data collection by participants, thus, alleviating the need for staff home visits which are expensive, time-consuming and potentially intrusive. In comparing our criterion validity indices to previous research, kappa, sensitivity, and specificity values appear to be similar to two previous studies reporting these values [8,9], and substantially higher than those reported by Marsh and colleagues [10]. Raynor and colleagues [2] are the only previous investigators to demonstrate significant criterion validity of groupings of high-fat and low-fat foods by showing significant correlations between reports of two adults living in the household. Our study examined several validity indices regarding staff and participant reports of regular fat and reduced fat versions of dairy, added fats, frozen and prepared desserts, and savory snacks and demonstrated substantial validity as well, and extended the previous work by examining subgroups of foods within major categories (e.g., cheese within the dairy category) rather than grouping all low-fat foods together. However, it should be noted that, although the comparison of staff and participant responses is an accepted practice for measuring criterion validity, this testing does not attest to the inventory's capture of all relevant foods, nor does it eliminate the possibility that participants may have altered their responses since the research staff were in their homes.

Although all of the HFI major food groups and many of the food subgroups showed substantial criterion validity, several of the food subgroups did not perform as well and deserve mention. In particular, the reduced-fat frozen dessert, regular fat prepared dessert, and white bread categories had lower than desired criterion validity. Our findings regarding lower validity for prepared desserts is similar to that found in previous research [8]. Several anecdotal observations early in the study indicate that the wide variety of dairy, soy and other frozen desserts available in the marketplace may make it difficult to assess nutrient and fat content. Similar observations were made for prepared desserts. In addition, the proliferation of whole grain white breads and light wheat breads available today confuse participants and staff alike when categorizing bread types.

Our findings regarding construct validity are similar to those reported by Raynor and colleagues [2]. In the present study, all of the correlations between the HFI major food categories and DHQ servings and nutrients were statistically significant in the expected direction. Several correlations between the HFI major food categories and child reported servings and nutrients were significant but attenuated in comparison to those of their parents. Perhaps it should be expected that food availability and intake would be more similar from the same informant (in this case parents) either because he/she purchases foods he/she prefers and eats. Another potential explanation for the poorer construct validity for the youth is that the youth's dietary intake was assessed using 24-hour recalls while the parents completed a food frequency that assessed usual food intake over a longer period of time. Further, our findings that an obesogenic food availability score for the household is significantly and positively associated with energy intake of both parents and adolescents indicate that high fat foods available in the home and captured on the inventory are potentially good starting points for public health messages for healthful eating.

The use of previous instruments and literature associated with energy consumption to determine the selection of foods for the instrument makes the home food inventory useful for many purposes. Previous home food availability measures were developed for specific study objectives such as fruit/vegetable consumption or foods associated with cancer. The broader selection of foods in our inventory increases its utility in nutrition- and obesity-based intervention programs.

There are several limitations that should be noted when interpreting the results of the present study. The present study did not assess test-retest reliability and therefore cannot address consistency of foods available in homes over time. However, consistency of foods may be less of an issue given that Raynor and colleagues [2] conducted two-week test-retest reliability of the absolute number of high-fat and low-fat foods and showed substantial stability. We also did not assess time since last shopping trip which could have influenced the home availability of perishable items or preferred foods which may be consumed more readily [6]. However, our significant correlations between the HFI scores and the DHQ food servings and nutrients indicate that perhaps this potential confounder was not influential. Another potential limitation is that the new home food inventory does not assess quantity; participants either check "yes" if the food is present in the home or "no" to indicate that the food is not present in the home. Accordingly, a household may "score" high on the number of fruits and vegetables even when quantity is limited or low on the number of fruits and vegetables when quantity is high for only a few foods. In addition, the list of foods is not an exhaustive list of all possible foods contributing to the obesity epidemic; however, in selecting foods to be included on the inventory, we balanced the number and types of foods with response burden. Our goal was to create an inventory that was simple and quick to complete. Attempting to collect data on more foods, quantities of foods, or more specifics about foods such as brand, would have impacted the time and complexity of completing the inventory and added to response burden. Moreover, our construct validity testing indicated that the foods measured on the inventory were significantly associated with energy intake from other measures, suggesting that we have captured a significant amount of the variation in the adult diet. Furthermore, the sample used for construct validity (Sample 2) over represented educated, Caucasian adults and findings may not generalize to less educated or minority populations.

The present study had several strengths. It is one of the few measures of the home food environment that has undergone criterion and construct validity testing, and it also has content validity for a broad range of foods that may be useful for assessing the obesogeneity of the home food environment. In particular, the criterion validity in which staff visited participants' homes to assess food availability was strong as was the construct validity between the HFI and the adult dietary intakes.

## Conclusion

This new home food inventory is a valid and participant-friendly tool to assess foods in the home, and may be useful for community-based behavioral nutrition and obesity prevention research. The inventory builds on previous measures by including a wide range of foods (both healthful and less healthful) rather than foods targeted for a specific intervention, as well as both perishable and nonperishable foods. Thus, the inventory may be useful in studies examining the contextual influences on obesity, weight gain, and nutritional intake. It might also be helpful in studies determining appropriate intervention strategies for individual households or might identify targets for public health messages.  Moreover, the present study findings indicate that the inventory has both construct and criterion validity, validity indices not typically assessed for the same instrument.

## Competing interests

The authors declare that they have no competing interests.

## Authors' contributions

JF conducted the literature review, drafted the items for the Home Food Inventory (HFI), trained staff, collected data for field testing and criterion validity testing, conducted analyses of the pilot data, and drafted the manuscript; MN and LL assisted in finalizing items for the HFI, assisted in scale score development, and edited the manuscript; SM trained staff, coordinated and collected data, and edited the manuscript; CH and KP assisted in scale score development and conducted data analyses. All authors have given final approval of this manuscript.

## Supplementary Material

Additional file 1 A table reflecting which foods are included in each food group/subgroup. Click here for file
