# Supplementary material for: The validation of a home food inventory
Source: Int J Behav Nutr Phys Act. 2008 Nov 4;5:55. doi: 10.1186/1479-5868-5-55 (PMC2587472; doi:10.1186/1479-5868-5-55)
Supplement: Additional file 1 — A table reflecting which foods are included in each food group/subgroup. [file 1479-5868-5-55-S1.doc]

# A table reflecting which foods are included in each food group/subgroup

| **Category** | | | **Sub-Category** | **Items** |
| --- | --- | --- | --- | --- |
| Dairy | Cheese | | Reg fat cheese | Shredded or block cheese, sliced cheese, ricotta or cottage cheese, cream cheese, Cheez Whiz, Velveeta, canned cheese or other similar cheese |
|  |  |  | Red fat cheese | Shredded or block reduced-fat cheese, sliced reduced-fat cheese, string cheese, mozzarella cheese, reduced–fat ricotta or cottage cheese, reduced-fat cream cheese or Neufchatel |
|  | Milk & other dairy drinks | | Regular milk | Whole milk |
|  |  |  | Red fat milk | Skim milk, 1% or 2% low fat milk, chocolate or flavored milk, reduced-fat yogurt drinks |
|  | Yogurt | | Regular yogurt | Regular yogurt |
|  |  |  | Red fat yogurt | Reduced-fat yogurt |
|  | Other dairy | | Regular dairy | Half and half, whipping cream or heavy cream, sour cream or cheese dips |
|  |  |  | Red fat dairy | Reduced-fat sour cream or low fat sour cream/cheese dips |
| Added fat | Regular fats | | | Butter, margarine, olive oil, vegetable oil, seed oil, lard, shortening, dressing |
|  | Light fat/fat substitutes | | | Light butter, light margarine or butter substitute, light/reduced fat dressing, light/reduced fat mayonnaise, Miracle Whip or other sandwich spread |
| Vegetables | All vegetables | | | Potatoes, asparagus, beets, bell peppers, broccoli, cabbage, cauliflower, carrots, celery, corn. cucumbers, green beans, lettuce, mushrooms, peas, spinach/other greens, squash, sweet potatoes, tomatoes, mixed vegetables |
|  | Vegetables without potatoes | | | Same as above with no potatoes |
| Fruit | | | | Apples, apple sauce, apricots, avocado, bananas, blueberries, cranberries, dates, grapes, grapefruit, kiwi, lemons or limes, mango, melons, mixed fruit, nectarines, oranges, pears, peaches, pineapple, plums, prunes, raisins, raspberries, strawberries, tangerines/clementines |
| Protein | High fat & processed meat | | | Bologna, salami, summer sausage, pepperoni, bacon, breakfast sausage, hot dogs, bratwurst, polish sausage |
|  | Other protein | | | Chicken/turkey, beef, pork, lamb, tofu, seitan, tempe, textured vegetable protein, veggie burger, fish, shellfish, lentils, beans, peanut butter, eggs, sliced turkey, sliced ham or roast beef |
| Frozen Desserts | Regular frozen desserts | | | Ice cream, frozen treats |
|  | Reduced fat frozen desserts | | | Reduced-fat ice cream, frozen yogurt, frozen treats made with ice milk, frozen yogurt, sherbet, or sorbet, frozen fruit juice bars |
| Microwavable or Quick-Cook Frozen Foods | | | | Pizza, Hot Pockets, pizza rolls or bagel snacks, burritos, chicken nuggets, French fries or tater tots, egg rolls, ramen noodles |
| Bread | | Wheat bread | | Wheat bread or rolls, English muffins, bagels, tortillas, pita bread |
|  |  | White bread | | White bread or rolls, English muffins, bagels, tortillas (flour or corn), pita bread, croissants |
| Prepared Desserts | | Regular prepared desserts | | Cookies, cake/cupcakes, muffins, brownies/bars, other snack cakes, pastry, sweet rolls, donuts |
|  |  | Reduced fat prepared desserts | | Reduced-fat cookies or cakes/cupcakes |
| Chips, Crackers and Other Snack Foods | | Regular snacks | | Whole grain snack crackers, snack crackers, potato chips, corn chips, tortilla chips, cheese curls or puffs, bagel chips, popcorn, peanuts, cashews or other nuts, granola bars, sports bars |
|  |  | Reduced fat snacks | | Reduced-fat snack crackers, potato chips, tortilla chips, cheese curls or puffs, bagel chips, graham crackers, pretzels, reduced-fat granola bars, sports bars |
| Dry Breakfast Cereal | | Whole grain | | Ready-to-eat cereals that are labeled “whole grain,” “whole wheat” or have at least 3 grams of fiber per serving |
|  |  | Low sugar cereal | | Ready-to-eat cereals that indicate on the nutrition label that they have  less than 6 grams of sugar per serving |
|  |  | High sugar cereal | | Ready-to-eat cereals that indicate on the nutrition label that they have  6 or more grams of sugar per serving |
| Beverages | | Regular beverages | | Regular soda pop, prepared iced teas or lemonade, sports drinks, 100% fruit juice, fruit drinks, soy milk or rice milk |
|  |  | Low sugar beverages | | Diet soda pop, prepared light iced teas or lemonade, bottled water |
| Candy | | | | Chocolate candy, hard candy, gummis, fruit rollups, fruit snacks or other fruit-based candy, chewy candy |
| Kitchen access | | Healthy kitchen access | | Fresh fruit, canned or dried fruit, fresh vegetables, reduced-fat snack crackers, pretzels, chips, and popcorn, diet soda, reduced-fat cookies, cake, cupcakes, muffins |
|  |  | Non-healthy kitchen access | | Regular snack crackers, pretzels, chips, and popcorn, dry cereal, bread or rolls, regular soda pop, candy, regular cookies, cake, cupcakes, muffins |
| Refrigerator access | | Healthy refrigerator access | | Skim milk, 1% or 2% milk, diet soda pop, bottled water, reduced-fat cheese, reduced-fat yogurt and yogurt drinks, fresh ready-to-eat vegetables and fruit |
|  |  | Non-healthy refrigerator access | | Whole milk, 100% fruit juice, fruit drinks/sports drinks, regular soda pop, regular cheese, regular yogurt |
